# An Unwarranted Assumption of Professional Duties

**Published:** 1919-03

**Authors:** Julio Endelman


### AN UNWARRANTED ASSUMPTION OF PROFESSIONAL DUTIES

BY JULIO ENDELMAN, D.D.S.

The advent of radiography in the practice of dentistry is responsible to a greater extent than any other measure for that improvement in the technique of dental operation and in the comprehension of pathological processes in the dental system, which has placed dentistry in the foreground of the medical and surgical specialties.

Radiography, on a par with anesthesia and aseptic surgery, has been sowing its benefactions everywhere; and the time is surely at hand when we would relinquish our professional duties rather than to be deprived of the diagnostic value of radiography in the treatment and filling of root canals. As a matter of fact, the pendulum has swung too far now in the direction of radiographic diagnosis to the detriment of those methods of clinical investigation which are being gradually neglected, if not abandoned, and without which we must insist a correct diagnosis can not be formulated in many cases, notwithstanding the availability of any number of radiographs. An optimism concerning the diagnostic efficiency of radiography born of that immutable law that bodies tend to move in the direction of least resistance, is responsible to a large extent for the present unsatisfactory status of dental diagnosis. Dentists as a class are gradually losing all sense of clinical diagnosis, leaning almost exclusively upon radiography with results that are not by any means invariably satisfactory. If radiographs could be depended upon, in all instances, to portray accurately the nature of pathological processes occurring within calcified tissues, no valid objection could be raised against its employment even to the abandonment of other methods of diagnosis. Radiography has a place in dental diagnosis, superseded in importance by perhaps no other method, but radiography alone can not be depended upon for a correct diagnosis in all cases, any more than clinical diagnosis can be relied upon to the exclusion of radiography. The happy combination of radiograhpy with clinical diagnosis leads to an accuracy of results which can not be attained by either of these methods separately, and should be practiced jointly whenever possible. To rely upon a radiograph for a diagnosis without the assistance of the history of the case, all subjective and objective symptoms, percussion and palpation, and other recognized procedures in diagnosis, is to place the patient's physical welfare at the mercy of the inaccuracies of the X-ray machine. Not infrequently dental diagnosticians find it necessary to secure as many as six radiographs of a single area before becoming satisfied that a fairly accurate portrayal of intraosseous conditions has been secured, and even then a diagnosis is withheld until a clinical examination is carried out. But, as against this, we have the commercial radiographer who, on the basis of perhaps only one picture and with no training whatsoever in the fundamental and special subjects of dentistry, ventures out with diagnoses of questional accuracy and dangerous possibilities. Securing a radiograph and interpreting it are two entirely different things, each requiring a different kind of knowledge, and *ipso facto* the ability to secure a radiograph does not presuppose the knowledge that is necessary in order to make a radiographic interpretation based on the anatomy of the area involved, and the nature of the pathological processes at work. We have had radiographs submitted to us with a written diagnosis (?) appended to them, prepared by men who possess no warrant, legal or moral, for venturing into fields unknown to them. And this adverse criticism is directed not alone at the radiographer who is not a dentist or a physician, but with equal force at those physicians who, unfamiliar with pathological processes in the dental system, diagnose abscesses where none are to be found, overlook peridental infections where it is obvious they are raging, give patients a clean bill of health as far as the mouth is concerned, even though an extensive chronic gingivitis may be present, or order the wholesale extraction of teeth for reasons unsupported by facts. The average physician has not the necessary dental educational qualifications to justify any assumption by him of the role of dental diagnostician. As between the radiographer without medical or dental training and the physician radiographer with a smattering of dental knowledge, we would not hesitate in choosing the former. He is the less dangerous of the two.

The time has come to call a halt to a practice,—to an assumption of professional duties as dangerous as it is reprehensible, by those who evidently do not know that for the purpose of acquiring the ability to diagnose and correct pathological processes or abnormalities in the mouth, dental colleges offer courses of instruction covering a period of not less than four years, to those possessed of the necessary preliminary educational requirements and moral qualifications. Dentists with a sufficient amount of training in dental pathology and allied subjects are the only ones justified in formulating diagnosis of dental conditions.—Editorial in *Pacific Dental Gazette.*

[May it not be true also that the practice of taking and studying radiograms may lead to closer clinical diagnosis by other means, and so lead us to more careful and systematic examination of all abnormal conditions?—*Editor,* REGISTER.]

